# Relationship of anxiety and burnout with extrasystoles in critical care nurses in Turkey

**DOI:** 10.12669/pjms.321.8407

**Published:** 2016

**Authors:** Yildiz Denat, Serap Gokce, Hasan Gungor, Cemil Zencir, Cagdas Akgullu

**Affiliations:** 1 Yildiz Denat, PhD, Assistant Professor, Adnan Menderes University, Aydin School of Health, 09100, Aydin, Turkey; 2 Serap Gokce, PhD, Research Assistan, Adnan Menderes University, Aydin School of Health, 09100, Aydin, Turkey; 3 Hasan Gungor, MD, Associate Professor of Cardiology, Adnan Menderes University Department of Cardiology, 09100, Aydin, Turkey; 4 Cemil Zencir, MD, Assistant Professor of Cardiology, Adnan Menderes University Department of Cardiology, 09100, Aydin, Turkey; 5 Cagdas Akgullu, MD, Associate Professor of Cardiology, Adnan Menderes University Department of Cardiology, 09100, Aydin, Turkey

**Keywords:** Anxiety, Burnout, Critical Care, Extrasystoles, Nursing

## Abstract

**Objective:**

To determine the relationship between levels of anxiety and burnout and prevalence of atrial extrasystoles (AESs) and ventricular extrasystoles (VESs) among critical care nurses.

**Methods:**

The sample of study included 51 nurses who worked in the intensive care units of a university hospital located in western Turkey. Beck’s Anxiety Inventory and the Maslach Burnout Inventory were used in the study.

**Results:**

The mean emotional exhaustion score of the nurses was 14.68±6.10, the mean personal accomplishment score was 19.19±7.08, the mean depersonalization score was 5.31±3.84 and the mean anxiety score was 12.37±11.12. The rates of VESs and AESs detected in the critical care nurses were 21.6% and 35.3%, respectively. No relationship was found between levels of anxiety and burnout and the prevalence of AESs and VESs among the critical care nurses. A positive correlation was found between personal accomplishment scores and numbers of VESs (r= 0.693, p=0.001) and AESs (r= 0.700, p= 0.001).

**Conclusion:**

In the present study, there were low mean scores of burnout and anxiety among nurses working in intensive care units. No relationship was found between levels of anxiety and burnout and the prevalence of AESs and VESs among nurses who work in intensive care units. It was found that the people feeling more personal accomplishment have more VES or AES. The prevalence of AESs and VESs among the critical care nurses suffering from burnout and anxiety may be studied in the future studies.

## INTRODUCTION

Burnout syndrome and anxiety are common, important problems for health workers, which have received increasing attention during recent years.^1^,^2^ Burnout has been defined as a particular type of work stress in professionals who provide services to people, which results from the difficult and emotionally charged relationships between providers and receivers of care.^3^ Meanwhile, anxiety is a diffuse and usually vague sense of apprehension, accompanied by various unpleasant physical sensations. Some studies emphasize that that nurses are at a high risk of developing anxiety and that nurses try to cope with chronic stress they are being exposed to by developing defense mechanisms and sometimes these defense mechanisms and anxiety/depression experiences reach pathological dimensions, which may result in the development of burnout syndrome. It has been reported that sometimes these defense mechanisms and anxiety reach pathological dimensions in their lives and may lead to the development of burnout syndrome.^4^-^6^ In a study by Turnipseed^7^, it was found that anxiety and burnout were related.

Burnout has been defined as a syndrome which includes emotional exhaustion, depersonalization and a perceived reduction in personal accomplishment. Burnout syndrome has a significant effect on the health and job efficiency of nurses working in departments with high levels of stress.^8^,^9^ Many studies have demonstrated that burnout is high among critical care nurses and report their symptoms of burnout.^9^-^13^

Research to date has shown that burnout has a negative effect on quality of life and mental health and that burnout has a negative effect on physical health and may be accepted as a risk factor for physical diseases and morbidity.^14^ Other evidence supports a relationship between burnout and cardiovascular risk factors such as dislipidemia and other metabolic syndrome components.^14^-^17^ In a study by Toker et al.,^15^ it was found that burnout was associated with a 1.4 times greater risk of coronary heart disease and that those who experienced higher levels of burnout had a higher risk (HR=1.79). Based on this evidence, it has been suggested that burnout may be a risk factor for coronary heart disease.^16^ It can be seen that the cardiac health of nurses, particularly critical care nurses, who work in stressful environments, may be at risk due to their high experience of burnout and anxiety. Therefore, in addition to examining cases of anxiety and burnout in critical care nurses, close monitoring of their cardiovascular health and taking the necessary precautions at an early stage is important both to increase the quality of care and to improve the health of nurses working in these departments.

Arrhythmias, especially atrial and ventricular arrhythmias, are generally considered benign in healthy individuals without structural heart disease. However, in cases like these, the evaluation and treatment of secondary causes such as stress, anxiety, anemia, hyperthyroidism, hypoxia, infection and fever is generally recommended.^18^

In clinical practice, these phenomena, which include premature heartbeats, are investigated with regards to stress. However, the number of studies evaluating the relationship between levels of anxiety and burnout and extra atrial and ventricular heartbeats in healthy hearts is limited. A study of 1114 patients in China found a relationship between anxiety symptoms and prevalence of extra ventricular heartbeats.^19^ However, no studies have been found which aim to investigate burnout, anxiety, cardiovascular symptoms and the relationship between them in critical care nurses, or Turkish critical care nurses in particular. This study aimed to investigate the relationship between levels of anxiety and burnout and the prevalence of atrial extrasystoles (AESs) and ventricular extrasystoles (VESs) among critical care nurses.

## METHODS

This study was conducted in the intensive care units of a university hospital located in western Turkey. The study sample consisted of 51 critical care nurses who were not diagnosed with structural heart disease, anemia, hyperthyroidism or asthma, had no active infection or fever, and was not using antidepressants or antiarrhythmic agents when they were included in the study.

Data were collected using an information form, Beck’s Anxiety Inventory (BAI) and the Maslach Burnout Inventory (MBI).^3^ One section of information form included demographic characteristics, educational level, information about working conditions and levels of satisfaction with the intensive care unit. The other section included records of their cardiac investigations.

Beck’s Anxiety Inventory (BAI) was developed by Beck et al.^20^ and Turkish reliability and validity studies of the scale were made by Ulusoy et al.^21^ It is a Likert-type scale with 21 items; each item is rated between 0 and 3 based on the response options. Higher total scores obtained from the inventory indicate the severity of the anxiety that the individual experiences.^20^-^21^ In our study, the Cronbach’s alpha reliability coefficient for the Anxiety Inventory was 0.94.

Maslach Burnout Inventory (MBI) was developed by Maslach and Jackson.^3^ It was first translated into Turkish and its reliability recalculated by Ergin and Cam.^22^-^23^ The scale consists of 22 questions. The scale measures three components of burnout; emotional exhaustion (EE), depersonalization (DP) and personal accomplishment (PA). High scores on the EE or DP subscales indicates burnout, as low scores on the PS subscale. In the present study, Cronbach’s alpha reliability coefficients for EE, DP, and PA were 0.85, 0.80, and 0.91, respectively.

All echocardiographic (ECHO) studies were obtained using a Vivid 5 (General Electric, Waukesha, WI, USA) with a 3.5 MHz probe for transthoracic exams. Each nurse was examined in terms of heart structure using ECHO. Each nurse was fitted with a Holter ECG monitor on the morning of a workday before they started work. The ECG Holter monitoring continued as long as they worked in the intensive care unit and during the night of that workday and was completed in 24 hours. All participants were evaluated by Holter ECG only one time and BAI and MBI was applied same day with Holter ECG. The study was approved by the the Ethics Committee of the Aydin Adnan Menderes University Clinical Research.

All analyses were carried out using the SPSS 16.0. Descriptive data were presented as numbers and percentages. Normally distributed data were analyzed using the Kolmogorov Smirnov test. Since the data were non-normally distributed, the Mann-Whitney U test was used to detect any significant differences between nurses with and without AESs/VESs in terms of mean Burnout and Anxiety scores; Spearman’s correlation analysis was used to determine correlations between the cardiac findings and burnout and anxiety. The statistical significance was accepted at the level of p <0.05.

## RESULTS

The mean age of nurse was 29.09±6.26 years. 86.3% of the nurses were female and 76.5% of them held a bachelor’s degree. 78.4% had work experience of 1 to 10 years, 74.5% were permanent staff, 92.2% were shift workers, 56.9% were working 40 hours per week, and 76.5% were responsible for the care of 2 or 3 patients during working hours. When asked their opinions of their job and working conditions, 62.7% stated that they had willingly selected their profession and 51% would not consider changing their job. 78.4% stated that they were willingly working in the intensive care unit, 78.4% declared that they believed they were competent in patient care and 70.6% were partially satisfied with their work life (Table-I).

**Table-I:** Caracteristics of nurses related to socio-demographic characteristics, professional life and work condition.

Socio-demographic characteristics	N (%)
*Gender*	
Female	44 (86.3)
Male	7 (13.7)
*Education*	
Vocational school degree	12 (23.5)
Bachelor’s degree	39 (76.5)
*Workplaces*	
Medical intensive care unit	23 (45.1)
Surgical intensive care unit	28 (54.9)
*Work experience*	
1-10 years	40 (78.4)
11-20 years	11 (21.6)
*Staff*	
Yes	38 (74.5)
No	13 (25.5)
*Shift workers*	
No	4 (7.8)
Yes	47 (92.2)
*Working hours per week*	
40 hours	29 (56.9)
48 hours	22 (43.1)
*The number of patients to be responsible during working hours*	
2-3 patients	39 (76.5)
4-6 patients	12 (23.5)
*Did you choose your profession willingly?*	
Yes	19 (37.3)
No	32 (62.7)
*Do you think changing your profession?*	
Yes	25 (49.0)
No	26 (51.0)
*Do you work with your own willingly in intensive care unit?*	
Yes	40 (78.4)
No	11(21.6)
*Are you competent in patient care?*	
Competent	40 (78.4)
Partially competent	11 (21.6)
*Are you satisfied with your work life?*	
Satisfied	15 (29.4)
Partially satisfied	36 (70.6)

In the analysis of burnout and anxiety scores, the mean EE score was 14.68±6.10, mean PA score was 19.19±7.08, mean DP score was 5.31±3.84, and mean anxiety score was 12.37±11.12. ECHO findings of all participants were normal. Holter ECG monitorings of participants were evaluated. VESs were detected in 21.6% and AESs in 35.3% of the critical care nurses participating in the study.

No statistically significant difference was found between the burnout scores of nurses with and without VESs (p>0.05) (Table-II). No statistically significant difference was found between the burnout scores of nurses with and without AESs (p>0.05). The statistical analysis did not reveal any significant difference between the mean anxiety scores of nurses with and without AESs (p>0.05) (Table-II).

**Table-II:** Distribution of burnout and anxiety score means in terms of the presence atrial and ventricular extrasystole.

Cardiac Finding	Burnout Scale Scores			Anxiety Score

	Emotional Exhaustion	Personal Accomplishment	Depersonalization	Mean±SD

	Mean±SD	Mean±SD	Mean±SD	
*Ventricular extrasystole*					
Detected	16.90±6.23	20.8 ±4.28	6.00±4.49	15.09±13.69
Not detected	14.07±6.00	18.75±7.66	5.12±3.68	11.62±10.38
U^†^/p*	U=156.50, p=0.14	U=195.00 p=0.56	U=197.00, p=0.59	U=189.00, p=0.47
*Atrial extrasystole*				
Detected	14.83±6.61	19.61±7.25	4.83±4.06	11.16±11.55
Not detected	14.60±5.91	18.96±7.09	5.57±3.75	13.03±11.00
U^†^/p*	U=283.00, p=0.78	U=274.00, p=0.64	U=262.50, p=0.49	U=266.50, p=0.54

† U; Mann-Whitney U Test.

* p<0.05 is significant.

In the assessment of the relationship between the cardiac findings and burnout and anxiety scores of the nurses, positive correlation was found between the PA sub dimension of burnout scores and number of VESs and AESs (p<0.001) (Table-III).

**Table-III:** Relationship between the cardiac findings and burnout and anxiety scores of nurses.

Cardiac Finding	Burnout Scale Scores						Anxiety Score	

	Emotional Exhaustion		Personal Accomplishment		Depersonalization		r	p

	r^††^	p	r	p**	r	p		
Ventricular extrasystole	0.048	0.888	0.693	0.001	0.005	0.989	0.100	0.769
Atrial extrasystole	-0.067	0.784	0.700	0.001	-0.219	0.368	0.001	0.997

†† r: Spearman’s correlarion analysis.

** p<0.01 is significant.

## DISCUSSION

No relationship was found between levels of anxiety and burnout and prevalence of AESs and VESs among nurses working in intensive care units. A positive correlation was found between the PA scores of the nurses participating in the study and the number of VESs and AESs. In the present study, there were low mean scores of EE and depersonalization and moderate scores of PA among nurses working in intensive care units.

Many studies on burnout in critical care nurses have revealed that nurses experience a moderate level or high level of burnout and have found that half of them show the symptoms of burnout.^14^-^17^ Previous studies conducted in Turkey have revealed that critical care nurses experience a higher level of burnout than nurses who work in other departments.^13^,^14^,^23^,^24^ In a study conducted in intensive care units by Dizer et al.^13^, the EE, DP and PA scores of nurses were found to be higher than those in the present study, while in the study conducted in intensive care units by Ozden et al.^14^ the EE, DP and PA scores of critical care nurses were found to be similar to those in the present study. Studies have emphasized that many factors may influence burnout, including total years in the profession, working hours, shift work, the unit where the nurse works, being happy in relationships with superiors and other teammates, finding the job suitable for themselves, worries about the future, feeling unhealthy, personal life and financial difficulties.^3^,^25^

It has been emphasized that nurses are at high risk of developing anxiety, as are all other healthcare professionals.^4^-^6^ In a study conducted by Ebrinc et al.^4^ the anxiety level of critical care nurses was found to be higher than that of nurses working in some other departments. In the present study, however, mean anxiety scores were low.

In this study, both emotional exhaustion and anxiety scores were low since the study sample was young, work experience ranged from1 year to10 years, most were working 40 hours a week, more than half had willingly selected the profession and would not consider changing their job, most were willingly working in intensive care and most believed that they were competent in patient care and were partially satisfied with their work-life. The fact that the sample group of the study had these features and the study was conducted in the intensive care unit of only one hospital constitutes the study limitation.

VESs were detected in 21.6% and AESs in 35.3% of the critical care nurses participating in the study. No significant difference was found between the mean of burnout and anxiety scores of nurses with and without VESs/AESs. In the analysis of relationships between cardiac findings and burnout and anxiety scores, a positive correlation was found between PA scores and the number of VESs and AESs. This result signified that people feeling a more personal accomplishment had greater VESs or AESs. The relationship between behavioral characteristics and cardiovascular disorders has been pointed out in the medical literature. In the literature, it has been emphasized that a success oriented, industrious, attentive, competitive and impetuous personality structure in individuals might prepare the ground for psychophysiological damage and that these personality characteristics are among the causes of heart diseases.^25^,^26^

## CONCLUSIONS

No relationship was found between levels of anxiety and burnout and prevalence of AESs and VESs among nurses working in intensive care units. However, the results highlighted a positive correlation between the number of VESs and AESs among critical care nurses and scores on the PA subscale of burnout. Nursing/hospital Management must take an active role for preventing burnout. It is recommended to inform the intensive care nurses about the effect of the increase in the sense of personal success upon cardiac health and train them for raising their awareness on this subject. The effects of working conditions on cardiac health should be investigated, along with an assessment of levels of anxiety and burnout, and measures should be taken according to the results, in order to improve the health status of critical care nurses.

## References

[1] Muslu C, Baltaci D, Kutanis R, Kara IH (2012). Quality of life, anxiety and depression in nurses working at primary health care and hospitals. Konuralp Med J.

[2] Taze S (2008). Determining the exhausted level of the nurses working in emergency and intensive care unit. Thesis's result for master for Marmara University.

[3] Maslach C, Jackson SE (1986). MBI: Maslach burnout inventory; manual (2nd).

[4] Ebrinc S, Acikel C, Basoglu C, Cetin M, Celikoz B (2002). Anxiety, depression, job satisfaction, burnout and coping with stress in nurses of a burn unit: A comparative study. Anatolian J Psychiatry.

[5] Raggio B, Malacarne P (2007). Burnout in intensive care unit. Minerva Anestesiol.

[6] Kaliterna LL, Prizmic LZ, Zganec N (2004). Quality of life, life satisfaction and happiness in shift- and non- shiftworkers. Rev Saude Publica.

[7] Turnipseed Dl (1998). Anxiety and burnout in the health care work environment. Psychological Reports.

[8] Lewandowski CA (2008). Organizational factors contributing to worker frustration: The precursor to burnout. J Sociogoly Soc Welfare.

[9] Kacmaz N (2005). Burnout syndrome. J Istanbul Uni Faculty Med.

[10] Poncet MC, Toullic P, Papazian L, Kentish-Barnes N, Timsit J, Pochard F (2007). Burnout syndrome in critical care nursing staff. Am J Respir Crit Care Med.

[11] Verdon M, Merlani P, Perneger T, Ricou B (2008). Burnout in a surgical ICU team. Intensive Care Med.

[12] Kranikola MNK, Papathanassoglou EDE, Mpouzika M (2012). Burnout syndrome indices in Greek intensive care nursing personnel. Dimens Crit Care Nurs.

[13] Dizer B, Iyigun E, Kilic S (2008). Determining the burnout levels of intensive care nurses. Turkish J Intensive Care Nurs.

[14] Ozden D, Karagozoglu S, Yildirim G (2013). Intensive care nurses' perception of futility;job satisfaction and burnout dim *e* nsions. Nursing Ethics.

[15] Toker S, Melamed S, Berliner S, Zeltser D, Shapira I (2012). Burnout and risk of coronary heart disease: A prospective study of 8838 employees. Psychosom Med.

[16] Melamed S, Kushnir T, Shirom A (1992). Burnout and risk factors for cardiovascular diseases. Behav Med.

[17] Kitaoka-Higashiguchi K, Morikawa Y, Miura K, Sakurai M, Ishizaki M, Kido T (2009). Burnout and risk factors for arteriosclerotic disease: follow-up study. J Occup Health.

[18] Binici Z, Intzilakis T, Nielsen OW, Køber L, Sajadieh A (2002). Excessive supraventricular ectopic activity and increased risk of atrial fibrillation and stroke. Circulation.

[19] Liang JJ, Huang H, Yang B, Wan J, Tang Y, Bao MW (2012). A cross-sectional survey on the prevalence of anxiety symptoms in Chinese patients with premature ventricular contractions without structural heart disease. Chin Med J.

[20] Beck AT, Epstein N, Brown G, Ster RA (1988). An inventory for measuring clinical anxiety: psycho-metric properties. J Consul Clin Psychol.

[21] Ulusoy M, Sahin NH, Erkmen H (1996). Turkish version of the Beck Anxiety Inventory: Psychometric properties. J Cognit Psychother.

[22] Ergin C (1992). The adaptation of the burnout inventory to the physicians and nurses.

[23] Cam O (1991). The validity of the burnout inventory 7th National Psychology congress, scientific studies.

[24] Demir A (1995). An investigation on nurses'burnout levels and some factors affecting these levels.

[25] Tuncel B (1997). Personality that predisposed to heart disease. Istanbul Univ J Faculty Communication.

[26] Yazici H, Altun F (2013). Type-A behavior, gender, and job satisfaction: a research on instructors. Educational Sciences: Theory Practice.

